# An Infrequent Histopathological Diagnosis in a Prevalent Systemic Disease: A Case of Renal Sarcoidosis

**DOI:** 10.7759/cureus.66633

**Published:** 2024-08-11

**Authors:** Sónia Reis Santos, Ana Cunha Rodrigues, José Luís Melo Pereira, Marta Costa, Nelson Domingues

**Affiliations:** 1 Internal Medicine, Unidade Local de Saúde Viseu Dão-Lafões, Viseu, PRT; 2 Nephrology, Unidade Local de Saúde Viseu Dão-Lafões, Viseu, PRT

**Keywords:** hypercalcemia, corticosteroids, acute kidney disease, renal sarcoidosis, hypercalcaemia, acute interstitial nephritis

## Abstract

Sarcoidosis is a multisystemic granulomatous disease that results from an aberrant immunological response to certain antigens. Although pulmonary involvement predominates, renal involvement may also occur. We present the case of a 59-year-old woman with a recent diagnosis of hepatic sarcoidosis and a history of non-compliance with treatment who was admitted to the hospital for study of acute kidney injury. Renal dysfunction was assumed to be a result of acute interstitial nephritis, as revealed on renal biopsy, and alterations in glomerular hemodynamics due to hypercalcemia. Renal function recovered and serum calcium levels normalized with the introduction of corticosteroid therapy.

## Introduction

Sarcoidosis is a multisystemic, idiopathic granulomatous disease characterized histologically by the presence of non-caseating granulomas of giant multinucleated epithelioid cells, preferentially distributed in the lungs, mediastinum, lymph nodes, eyes, and skin [1,2]. Its pathophysiology has not been fully understood, but it is believed to involve an aberrant immune response resulting from an imbalance between effector and regulatory T cells with consequent macrophage polarization and granuloma formation in genetically and environmentally predisposed patients after exposure to a diverse set of antigens [3].

Pulmonary involvement is the most common condition associated with sarcoidosis (present in more than 90% of diagnosed patients), but any organ can be affected as disease progresses. The onset of clinical manifestations typically occurs between the second and fifth decades of life, with females being preferentially affected, and a global prevalence is estimated to be 10 to 160 per 100,000 inhabitants [3]. Although mostly it has a benign course with spontaneous resolution of its clinical manifestations [4], in around one-third of sarcoidosis patients, it can develop into a potentially serious chronic disease with a mortality rate of up to 5% [5].

According to systematic literature reviews, kidney involvement occurs in around 25% to 30% of patients and progresses to end-stage renal disease in 0.7% to 4.3% of them [2,3]. Although uncommon, renal involvement in sarcoidosis is clinically relevant not only because of the risk of progression to end-stage renal disease in the absence of adequate early treatment [6] but also because it may imply adjustments to the therapy used in extrarenal forms of the disease [4]. Renal sarcoidosis is most often underdiagnosed, which is due to not only low level of clinical suspicion but also the absence of changes in renal function or urinalysis at the time of presentation of the disease [6]. Several mechanisms of kidney damage have been described in sarcoidosis, including calcium metabolism disorders (24% to 27% of cases) with nephrolithiasis, nephrocalcinosis, and/or changes in glomerular hemodynamics induced by hypercalcemia; granulomatous interstitial nephritis (7% to 27% of patients); or, more rarely, glomerular involvement, particularly membranous nephropathy, minimal change disease, proliferative or crescentic glomerulonephritis, focal glomerulosclerosis, and even IgA nephropathy [1].

Its diagnosis is made by exclusion in the case of a patient with a suggestive clinical picture, concordant complementary examinations, and a confirmatory histopathological diagnosis, with evidence of characteristic lesions in at least one organ system [4].

This clinical case aims to demonstrate the integral diagnostic approach in the assessment of a patient with acute kidney injury, whose substrate is a systemic immunological disease.

## Case presentation

A 59-year-old woman, autonomous in her activities of daily living, was admitted to the Multipurpose Emergency Service (MES) with nausea, generalized tremors, and a week-long notion of decreased urine output. She denied fever, eye, skin, respiratory, gastrointestinal, or osteoarticular complaints. Her pathological history included hypertension, scleroderma affecting the skin, and hepatic sarcoidosis, with a recent histological diagnosis of granulomatous hepatitis after years of follow-up due to altered liver function tests and the exclusion of toxic and infectious etiology (brucellosis, hepatitis A/B/C, human immunodeficiency virus, syphilis, cytomegalovirus, toxoplasma, and Epstein-Barr virus). The patient had a poor adherence to sarcoidosis therapy due to apparent intolerance/skin reactions to previous treatment with corticosteroids, azathioprine, ursodeoxycholic acid, and hydroxychloroquine.

On objective examination, she was apyretic and hemodynamically stable, with no alterations on cardiopulmonary auscultation and brief neurological examination. Plaque morphea lesions were identified in the submammary, abdominal, and lower limb regions. There were no signs of inflammation or joint swelling.

The study carried out on the MES showed normocytic and normochromic anemia (hemoglobin 11.5mg/dL), acute kidney injury (new-onset creatinine 2.3mg/dL, baseline creatinine 0.8mg/dL and urea 61mg/dL), hypercalcemia (ionized calcium 1.6mEq/L), and high SACE level (serum angiotensin-converting enzyme) of 157U/L. Renovesical ultrasound showed kidneys with normal morphology and topography, with a good preservation of sinus-parenchyma differentiation and no alterations to the excretory cavities or obvious images of lithiasis.

**Figure 1:**
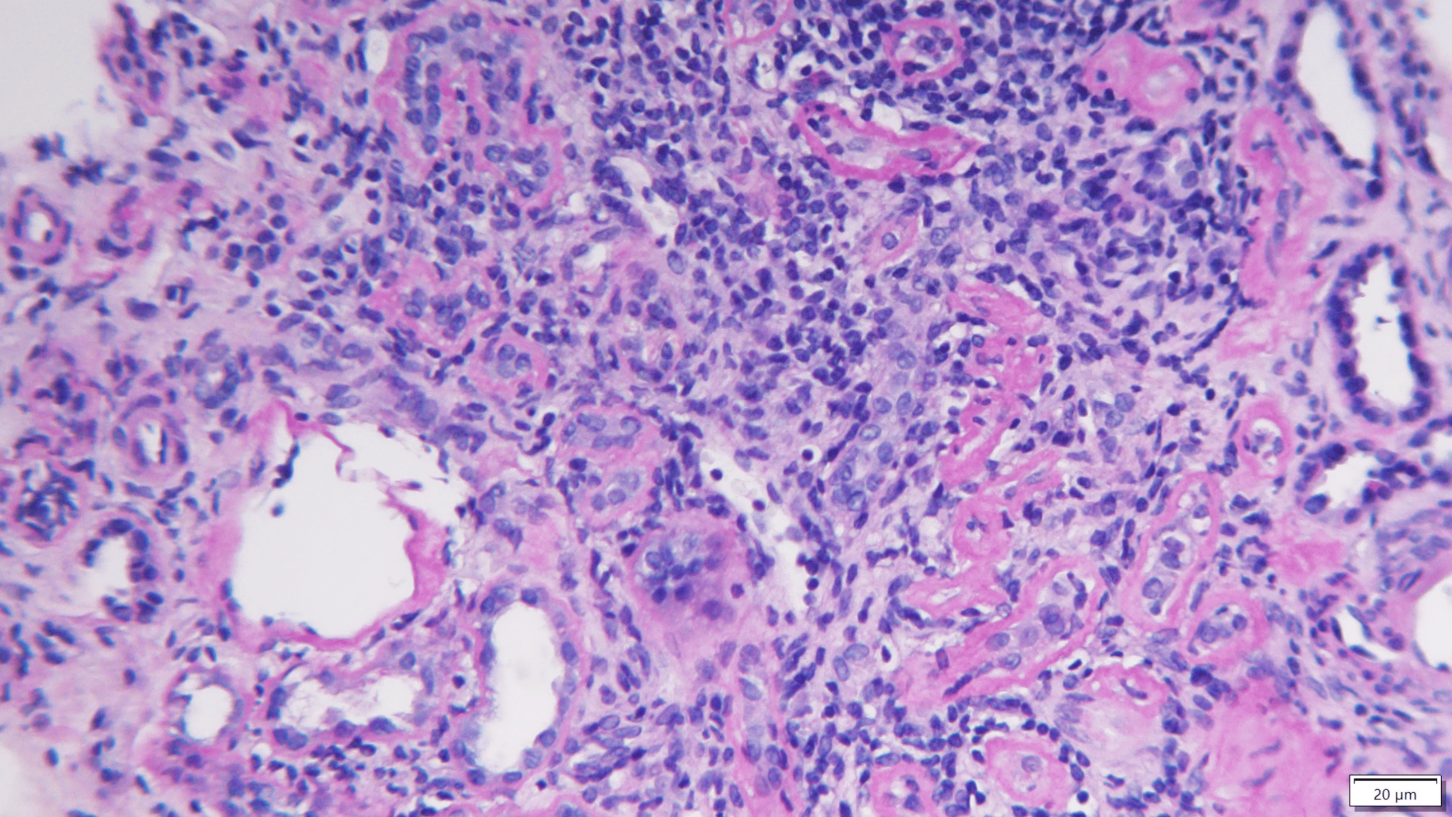
Kidney biopsy Interstitial infiltrate of mononuclear cells, compatible with acute interstitial nephritis.

She was therefore admitted to the Nephrology Ward under medical therapy to correct the hypercalcemia and for monitoring and studying the acute kidney injury in a patient with a history of systemic disease. Hypercalcemia persisted despite the measures instituted, and there was no improvement in renal function (creatinine 2.4mg/dL and ionized calcium 1.72 mmol/L). The complementary study was inconclusive (Tables 1, 2), and a renal biopsy was then performed. Faced with the most likely diagnostic hypotheses of acute kidney injury secondary to hypercalcemia and/or intrinsic kidney involvement due to sarcoidosis, after a multidisciplinary discussion with rheumatology, empirical corticosteroid therapy was proposed and she was started on methylprednisolone 0.5mg/kg/day. At the time of discharge, kidney function and hypercalcemia were stable, and she was referred to the nephrology and systemic autoimmune disease departments of Internal Medicine (given the absence of clinical/systemic and osteoarticular complications) under deflazacort 45mg/day, pantoprazole 20mg, and vitamin D supplementation (addressing her deficiency values), awaiting the results of the renal biopsy. Kidney histology confirmed the hypothesized etiologies. On light microscopy, an interstitial infiltrate of mononuclear cells was evident, compatible with acute interstitial nephritis (Figure 1). Acute tubular necrosis was present in 50% of the cortex observed, and tubular calcium phosphate crystals were also seen. Glomerulus and blood vessels were normal, and the immunofluorescence study was negative. The instituted therapy was maintained.

**Table 1:** Initial analytical evaluation of acute kidney injury in the nephrology ward Anti-dsDNA Ac, anti-double stranded DNA antibodies

Analytical parameter		Laboratory results	Reference ranges
Urea		59 mg/dL	19–49 mg/dL
Creatinine		2.4 mg/dL	0.5–1.2 mg/dL
Sodium		137 mEq/L	135–145 mEq/L
Potassium		4.5 mEq/L	3.5–5.0 mEq/L
Chlorine		100.5 mEq/L	95.0–110.0 mEq/L
Total calcium		6.2 mg/dL	4.2–5.1 mg/dL
Ionized calcium		1.72 mmol/L	1.16–1.31 mmol/L
Phosphorus		3.3 mEq/L	2.8–4.1 mEq/L
Albumin		3.8 g/dL	3.5–5.0 g/dL
Parathormone		6.00 pg/mL	18.50–88.00 pg/mL
Proteinuria (urine sample)		13.6 mg/dL	1.0–14.0 mg/dL
Urine sediment	Leukocyturia	23/µL	1.0–10.0/µL
	Erythrocyturia	9/µL	1.0–24.0/µL
Anti-glomerular basement membrane antibody		0.9 U/mL	0–10 U/mL
Anti-dsDNA Ac.		12.0	0.0–15.0

**Table 2:** Complementary study carried out during the diagnostic process ANA, antinuclear antibodies; ENA, extractable nuclear antigen antibodies; C, complement

Analytical parameter	Results
Gamma interferon (QuantiFERON)	Negative
Wright's reaction	Negative
ANA	Positive (1/320) in a homogeneous pattern
ENA screen	Negative
C3, C4	Normal
Serum electrophoretic proteinogram and immunofixation	Exclusion of monoclonal pathology with evident polyclonal hypergammaglobulinemia

Two months after discharge from the hospital, the patient's renal function, calcium levels, and anemia had normalized (creatinine 0.9mg/dL, total calcium 4.9mEq/L with albumin 4.4g/dL, and hemoglobin 12.4g/dL), SACE (serum angiotensin-converting enzyme) was normal, and albuminuria was absent. Corticosteroid therapy was weaned after eight weeks when the disease became stable and after the introduction of azathioprine 75mg twice daily. The patient maintained follow-up in both specialties with stabilization of the disease activity and renal function at two years' follow-up under prednisolone 2.5mg and azathioprine 75mg bid, with no record of new organ systems being affected. Intercurrences included the development of diabetes secondary to corticosteroid therapy, requiring insulinization, and the occurrence of osteoporotic fractures requiring vertebroplasty.

## Discussion

Like any multisystemic disease, the spectrum of clinical manifestations associated with sarcoidosis is highly diverse, an aspect that adds difficulty to its proper and timely diagnosis, which most often begins after abnormalities are detected on a chest X-ray. In the absence of specific tests for sarcoidosis, the American Thoracic Society has defined the following three indispensable criteria for its diagnosis: the presence of clinical changes that are practically specific to the diagnosis (dyspnea, fatigue, nocturnal hyperhidrosis, hilar and peripheral adenopathies, erythema nodosum and lupus pernio, facial paralysis, arrhythmias, and congestive heart failure), the presence of non-caseating granulomas in at least one organ, and the exclusion of other alternative etiologies for granulomatous disease [3,5]. Despite the typical association of non-caseous and non-necrotizing granulomas with sarcoidosis, these are not pathognomonic of the disease, and infectious pathology, zoonoses (brucellosis, tuberculosis, leprosy, and histoplasmosis), neoplasms (breast, lungs, and Hodgkin's lymphoma), berylliosis, immunological diseases (Crohn's disease, IgG4 disease, primary Stevens-Johnson syndrome, and primary biliary cholangitis) and drug iatrogenesis (anti-inflammatories, allopurinol, fluoroquinolones, diuretics, and antiretrovirals) must be excluded [1].

Although its diagnosis is possible in the absence of pulmonary and ganglionic involvement [5], the presence of systemic manifestations favors the clinical suspicion of renal involvement by sarcoidosis [3] in patients with a decline in glomerular filtration rate, albuminuria of more than 300mg/24 hours, or alterations in the urine test (mostly sterile pyuria, hypercalciuria, and microhematuria). The definitive diagnosis depends on histopathological confirmation in a kidney biopsy [1].

In the clinical case presented, the etiology of acute kidney injury is multifactorial: in addition to the histological diagnosis of acute interstitial nephritis (AIN) without an alternative culprit besides sarcoidosis, there is the impact of hypercalcemia on acute tubular necrosis. Although calcium metabolism abnormalities represent the most prevalent cause of kidney dysfunction in sarcoidosis patients, granulomatous interstitial nephritis appears as the most typical histological finding, observed in 7% to 23% of kidney biopsies [4]. Nevertheless, similar to the case presented, granulomas are not seen in all biopsies of hypovolemia patients with sarcoid-associated interstitial nephritis.

In sarcoidosis and other granulomatous diseases, hypercalcemia comes from an increase in the concentration of the active form of vitamin D due to the action of the 1α-hydroxylase enzyme produced by granulomas, in a process independent of the normal negative feedback mechanisms, which culminates in an increase in intestinal calcium absorption, stimulation of osteoclast activity in bone resorption, and an increase in renal tubular calcium resorption [4]. Hypercalcemia leads to renal dysfunction by various mechanisms, such as renal hypoperfusion due to vasoconstriction of the afferent arteriole, hypovolemia as a consequence of sodium and water spoliation due to blockage of the Na-K-ATPase pump, dysfunction of urinary concentration mechanisms due to decreased sensitivity to antidiuretic hormone, and acute tubular necrosis resulting from intracellular calcium overload and obstruction of the renal tubules. Hypercalciuria (present in 40% to 62% of patients) therefore favors the development of nephrolithiasis and obstructive uropathy [1,4]. In the absence of adequate treatment, the perpetuation of hypercalcemia and hypercalciuria leads to progressive tubulointerstitial fibrosis and nephrocalcinosis, which are responsible for the progression of chronic kidney disease [4]. Although granulomatous interstitial nephritis represents the most characteristic histopathological pattern in patients with renal sarcoidosis, its diagnosis can be hampered by the absence or benignity of its clinical signs such as microscopic hematuria, mild proteinuria, or sterile pyuria associated with elevated serum creatinine and reduced glomerular filtration rate [7].

As with other forms of extrapulmonary sarcoidosis, when the kidneys are involved, the introduction of immunosuppressive treatment (mainly corticosteroid therapy) is crucial to halt progression to end-stage renal disease and avoid the need for renal replacement therapy suffices, either by regressing the inflammatory process or by correcting hypercalcemia [6]. The final response to the therapy is dependent on the initial degree of fibrosis in the histopathological study. During the treatment of these patients, attention should be paid to the development of complications inherent to prolonged corticosteroid therapy, namely secondary diabetes, osteoporosis, hypertension, and central obesity [4].

In the minority of patients who progress to end-stage renal disease, the results of renal replacement therapies, whether dialysis or transplantation, are similar to those seen for the other etiologies of renal disease. The risk of sarcoidosis recurring in the kidney graft is not negligible, and the likelihood is higher in patients undergoing the procedure after a short period of time since the last crisis. Sustained correction of hypercalcemia is fundamental in reducing the risk of recurrence [7].

## Conclusions

Despite the infrequent renal involvement in sarcoidosis, it is clinically significant and probably underdiagnosed in this multisystemic disease. Given the risk of organ dysfunction with the perpetuation of untreated disease, it is essential to screen for renal involvement when diagnosing and following up on patients with sarcoidosis by assessing urinary sediment, quantifying proteinuria, and monitoring renal function.

With this case, the authors want to emphasize the importance of sarcoidosis as a multi-organ disease and recommend that clinicians be aware of the systems involved in the disease when making the initial diagnosis, as the earlier the diagnosis and the start of appropriate therapy, the greater the likelihood of successful therapeutic interventions.
